# Can cost-effectiveness results be combined into a coherent league table? Case study from one high-income country

**DOI:** 10.1186/s12963-019-0192-x

**Published:** 2019-08-05

**Authors:** Nick Wilson, Anna Davies, Naomi Brewer, Nhung Nghiem, Linda Cobiac, Tony Blakely

**Affiliations:** 1 BODE³ Programme, Department of Public Health, University of Otago Wellington, Wellington, New Zealand; 2 Centre for Public Health Research, Massey University, Wellington, New Zealand; 3 Nuffield Department of Population Health, University of Oxford, Oxford, UK

**Keywords:** League table, Cost-effectiveness, Cost-utility, Health interventions

## Abstract

**Background:**

Doubts exist around the value of compiling league tables for cost-effectiveness results for health interventions, primarily due to methods differences. We aimed to determine if a reasonably coherent league table could be compiled using published studies for one high-income country: New Zealand (NZ).

**Methods:**

Literature searches were conducted to identify NZ-relevant studies published in the peer-reviewed journal literature between 1 January 2010 and 8 October 2017. Only studies with the following metrics were included: cost per quality-adjusted life-year or disability-adjusted life-year or life-year (QALY/DALY/LY). Key study features were abstracted and a summary league table produced which classified the studies in terms of cost-effectiveness.

**Results:**

A total of 21 cost-effectiveness studies which met the inclusion criteria were identified. There were some large methodological differences between the studies, particularly in the time horizon (1 year to lifetime) but also discount rates (range 0 to 10%). Nevertheless, we were able to group the incremental cost-effectiveness ratios (ICERs) into general categories of being reported as cost-saving (19%), cost-effective (71%), and not cost-effective (10%). The median ICER (adjusted to 2017 NZ$) was ~ $5000 per QALY/DALY/LY (~US$3500). However, for some interventions, there is high uncertainty around the intervention effectiveness and declining adherence over time.

**Conclusions:**

It seemed possible to produce a reasonably coherent league table for the ICER values from different studies (within broad groupings) in this high-income country. Most interventions were cost-effective and a fifth were cost-saving. Nevertheless, study methodologies did vary widely and researchers need to pay more attention to using standardised methods that allow their results to be included in future league tables.

**Electronic supplementary material:**

The online version of this article (10.1186/s12963-019-0192-x) contains supplementary material, which is available to authorized users.

## Background

Given the constraints on health sector resources and the large demand for expanded or new health sector interventions, it is important to determine which interventions get the best value for money out of the limited resources available. As part of this prioritisation, cost-effectiveness is an important criterion for policymakers to consider, although it should be considered alongside other issues such as health equity, the size of the health gain, the timing of health gains and costs, issues around intervention feasibility and public acceptability, etc.

Previous international work has generated league tables for intervention cost-effectiveness from studies in multiple countries [1–4], for specific countries (e.g., the USA [5] and Australia [6, 7]), and for specific topic domains such as cancer care [8] and dietary sodium reduction [9]. All of this work suggests that there are many cost-saving and cost-effective interventions that policymakers could adopt or enhance use of. Nevertheless, there are often method differences between studies that may limit the coherence of such league tables [10].

The country in our case study presented here (New Zealand (NZ)) is a fairly typical high-income country in which demand for health services is constantly increasing relative to available resources. However, New Zealand does have a single national agency that successfully constrains rising pharmaceutical prices (PHARMAC) and conducts cost-utility analyses as part of its decision-making processes. But elsewhere, in the health sector, there is no agency that routinely does such analyses and systematically prioritises health sector interventions. There is also no government-wide threshold for when gaining a quality-adjusted life-year (QALY) is deemed “cost-effective” or the relative importance of gaining QALYs in different populations as part of reducing health inequalities (given the health gaps between Māori (indigenous) and non-Māori in this country).

Despite this, a league table has been published of 21 pharmaceutical interventions in New Zealand [11]. This work reported one intervention that was cost-saving and 20 with cost-effectiveness ratios ranging from NZ$771 to $142,000 per QALY (with a median value of the latter group of $9290, and mean of $25,300 [with these values calculated by us]). Other New Zealand-specific league tables include one for 12 dietary sodium reduction interventions (of which all but one were cost-saving [12]) and one for five tobacco control interventions (all cost-saving [13]). We have also developed an online interactive league table [14] that encompasses all the interventions modelled by the Burden of Disease Epidemiology, Equity and Cost-Effectiveness Programme (BODE^3^) Programme (covering tobacco control, nutrition, injury prevention, cancer prevention, and cancer treatment and management). However, in this particular study, we aimed to identify if other published cost-effectiveness analyses (CEAs) performed for New Zealand by different research teams could be included in a single reasonably coherent league table.

## Methods

### Literature searches

We first aimed to identify published New Zealand-specific CEAs that meet all of the following criteria: (i) the study was published in a peer-reviewed journal in the time-period 1 January 2010 to 8 October 2017; (ii) the study results included one of the following metrics: a cost per QALY or life-year (LY) gained or a cost per disability-adjusted life-year (DALY) averted; (iii) the study involved New Zealand epidemiological or cost data (even if New Zealand data was part of a combined study with other countries); and (iv) the study was not an output of the BODE^3^ Programme, since results from this programme all use a standard methodology and are detailed in an online interactive league table [14] (with a journal article about this league table pending).

The specific aspects of the search strategy are detailed in Table 1 and examples of excluded studies are detailed in Additional file 1: Table A1.

**Table 1:** Search strategy used to identify cost-effectiveness studies relevant to New Zealand

Search strategy with text terms (using PubMed unless indicated otherwise and for the search period 1 January 2010 to 8 October 2017, articles with abstracts, human-only studies, and English language)	Total results	Additional articles meeting inclusion criteria (after the search in row 1)
Zealand AND (cost-effective OR cost-utility OR cost-benefit OR benefit-cost OR “economic evaluation”)	459	22 (but with 1 duplicating the results of another)
Zealand AND (QALY OR DALY OR life-year OR ICER)	138	0
Zealand AND “cost per”	56	0
Authors publishing on health economics and known to work/have worked in NZ in the past decade: Ashton T, Brown P, Cumming J, Edlin R, Green T, Hansen P, Harris J, Leung W, Milne R, O’Dea D, Scott H, Scott W, Sheerin I. (Combined with the search term: “Zealand”)	198	0
Tufts Medical Center Registry (searching for “Zealand”) (http://healtheconomics.tuftsmedicalcenter.org/cear4/SearchingtheCEARegistry/SearchtheCEARegistry.aspx)	37	0
Centre for Reviews and Dissemination (CRD) database, University of York (https://www.crd.york.ac.uk/CRDWeb/) with Abstract or full published health technology assessment articles (searching for “Zealand”)	115	0
Bibliography search of the selected articles (all CEAs and CBAs)	38	1

*CEAs* cost-effectiveness analyses, *CBAs* cost-benefit analyses, *DALY* disability-adjusted life-year, *ICER* incremental cost-effectiveness ratio, *NZ* New Zealand, *QALY* quality-adjusted life-year

### Threshold for cost-effectiveness

A cost-effectiveness threshold from a policymaker's perspective will vary depending on what (dis)investment opportunities are being considered and what new (or requirement to reduce) funding is available, at a given time for a given investment horizon. Therefore, funding agencies usually do not have an official threshold—including in New Zealand. Nevertheless, as a guide, we note that on occasions, New Zealand Government agencies have quoted a value from the New Zealand Treasury of $38,110 per QALY (e.g., as per a Ministry of Health Report [15]), which is close to the World Health Organization guidance [16] of the per capita gross domestic product (GDP) of a country per QALY gained, i.e., $45,000 in NZ$ 2011 [17]).

### Adjustment to NZ$ 2017

After abstracting the key information from each study, we then scaled the incremental cost-effectiveness ratio (ICER) values to 2017 NZ$ using the consumer price index (CPI) and purchasing power parity (PPP) (when translating from other currencies).

## Results

A total of 21 cost-effectiveness studies were identified and their key characteristics are summarised in Table 2 (with more detailed summary data and commentary in Additional file 1 Tables A2, A4, and A5 in the Additional file 1). Of these, the reported incremental cost-effectiveness ratios (ICERs) suggest that 19% had cost-saving interventions, 71% had main results that can be classified as cost-effective, and 10% were not cost-effective using New Zealand GDP per capita as a threshold. The median value (2017 NZ$) was $5080 per QALY/DALY/LY as per the values in Table 2 and taking the first figure if multiple values are shown.

**Table 2:** League table of the 21 New Zealand cost-effectiveness studies identified and published in the period 1 January 2010 to 8 October 2017 (ordered by decreasing cost-effectiveness, with additional details on each study in the Additional file 1: Tables A2, A4, and A5)

Study reference	Intervention*	Reported ICER (NZ$)**	ICER (NZ$ 2017)**
Cost-saving interventions			
Leung et al. [20]	Pedometer-based promotion in primary care versus time-based activity goals via green prescriptions	Cost-saving	Cost-saving
O’Keeffe et al. [21] and Scott et al. [22]	Diagnosis and treatment pathways for insomnia for a range of practitioners including: pharmacists, general practitioners (GPs), psychologists, other health professionals, and alternative health practitioners	Cost-saving	Cost-saving
Lew et al. [23]	Primary human papillomavirus (HPV) screening with partial genotyping in both unvaccinated women and cohorts offered vaccination	Cost-saving relative to current practice; One strategy (S2a): $20,600 per QALY saved in unvaccinated scenario; $9770 in vaccinated scenario (both compared to next best strategy) (2015 NZ$)	Cost-saving to 20,800 (for S2a strategy)
Friedman et al. [24]	Proposed national programme to prevent paediatric abusive head trauma (AHT, often known as “shaken baby syndrome”)	Cost-saving in most scenarios, i.e., where reduction in AHT is 30% or more and intervention cost is between $20 and $100 per new-born. However, some estimates were as high as $471,000 per QALY (2012 NZ$)	Cost-saving to 492,000
Cost-effective interventions			
Gander et al. [25]	Diagnosis and treatment pathways for obstructive sleep apnoea syndrome (OSAS) from GP level through to surgical intervention	$94 per QALY (2005 NZ$)	121
Lake et al. [26]	Campylobacter control in NZ poultry meat supply: interventions at all points from farm to consumer (as per the situation in 2005)	Range: from NZ$1200 per DALY (primary processing interventions) to NZ$43,400 per DALY (irradiation at primary processing stage) (2009 NZ$)	1360 to 49,300
Webb et al. [27]	A “soft regulation” national policy for dietary sodium reduction that combines targeted industry agreements, government monitoring, and public education (modelled on the UK programme)	I$989/DALY (using 2013 I$)	1480
Maddison et al. [28]	Improving exercise capacity and physical activity through a mobile phone / online intervention in addition to usual care, for people with ischaemic heart disease (IHD)	$2690 per QALY (for the 12 month timeframe) (2012 NZ$)	2810
Dalziel et al. [29]	A broad range of interventions to prevent neural tube defects (from targeted promotion of folic acid supplement to voluntary/mandatory folic acid fortification of the food supply)	$2700 per DALY for physician advice for supplement use; $6500 per DALY for a health promotion campaign for supplement use; (both targeted at women around the time of conception) (2006 NZ$)	3370 and 8120
Sopina and Ashton [30]	18 different cervical cancer screening combinations (e.g., based on usage of the HPV vaccine, screening interval length (3 or 5 years), etc.	$3560 to $10,200 per QALY (for a “no vaccine” base case comparison) (2009 NZ$)	4040 to 11,540
Panattoni et al. [31]	Treatment of acute coronary syndrome with prasugrel if the person is a carrier of the CYP2C19*2 allele (if not a carrier of this allele, the person gets treatment with clopidogrel)	$4480 per QALY when using prasugrel instead of clopidogrel; and $8700 per QALY (if the former is genetically guided) (2009 NZ$).	5080 and 9880
Simms et al. [32]	Strategies for screening for HPV in context of a nonavalent vaccine (“HPV9 vaccine”)	$5000 per LY saved for 5 screens per lifetime (for cohorts offered nonavalent vaccine) (2013 NZ$)	5170
Te Ao et al. [33]	Increasing the use of thrombolysis treatment for ischaemic stroke by increasing hospital presentations and / or increasing use of thrombolysis treatment in hospital	$6640 per QALY (lifetime) and $27,000 (first year) (2010 NZ$)	7380 and 30,000
Te Ao et al. [34]	Acute stroke units in NZ hospitals (as opposed to care on general wards)	$6750 per QALY (lifetime) and $42,800 per QALY (first year) for care in an acute stroke unit vs a general ward (2008 NZ$)	7960 and 50,500
Keall et al. [35]	Package of home modifications to reduce injuries from falls at home	$14,300 per DALY when just considering intervention costs, i.e., no cost offsets (2012 NZ$).	14,900
Milne et al. [36]	Long-term air humidification therapy plus usual care for people with moderate/severe COPD/bronchiectasis	$20,900 per QALY (mean) (2012–2013 NZ$)	21,600
Rush et al. [37]	A multicomponent through-school physical activity and nutrition programme (“Project Energize”)	Range from $22,200 to $30,400 per QALY (depending on age and ethnicity) (2011 NZ$)	24,100 to 33,100
Pinto et al. [38]	Knee/hip osteoarthritis (OA) treatment: manual therapy, exercise therapy, or both, plus usual care	Range from $26,400 per QALY (exercise therapy) to $149,000 (combined therapy) from the health system perspective (2009 NZ$)	30,000 to 169,000
Carrasco et al. [39]	Antiviral stockpiling for future influenza pandemics (relative to no stockpiling)	Approximately US$20,000 per QALY (for the most plausible scenario of 30% of misallocation of antivirals) (2010 US$)	33,200
Not cost-effective interventions			
Harris et al. [40]	Planned early start for dialysis treatment based on kidney function for patients with progressive chronic kidney disease.	72% of results indicated reduced health gain and increased costs. Only 0.3% of iterations gave a positive QALY at under $50,000 per QALY	Not estimated
Leung et al. [41]	Exercise counselling intervention to enhance smoking cessation	$451,000 per QALY (using 24 week follow-up) (2012 NZ$)	455,000

*The comparator is current practice/usual care unless otherwise specified (with more details in Table A4 in Additional file 1)

**All values are rounded to three meaningful digits

Table 3 shows additional details relating to the study methods including the variable discount rates (range 0 to 10%). Only 57% of the studies used a 3.0% or 3.5% discount rate (with 3.0% being the internationally recommended rate [18], but within New Zealand, 3.5% is recommended by the Pharmaceutical Management Agency (PHARMAC; the pharmaceutical and device funding agency for the New Zealand Government)). QALYs were used in 76% of the studies and around half (48%) used a lifetime time horizon. However, 24% used a very short time horizon of only 12 months (i.e., usually the length of the trial upon which the health economic analysis was based). Most studies used a health system perspective (86%), with the rest being a societal one (two studies had both). Only three of the studies included productivity losses, and none include greenhouse gas emissions, even though a number of interventions involved dietary changes and transport costs.

**Table 3:** Summary characteristics of the 21 studies with cost-effectiveness analyses for New Zealand in the period 1 January 2010 to 8 October 2017 (for the key interventions in each paper as shown in Table 2)

Study characteristic	Number of studies	% of all 21 studies
Key methods		
Discount rate of 0% or not stated (mainly 1-year trials)	6	29
Discount rate used includes 3.0% or 3.5%	12	57
Discount rate of 5% or 10%	3	14
Used QALYs	16*	76
Used DALYs	4	19
Used LYs	3*	14
Time horizon was lifetime	10	48
Time horizon was only 12 months	5	24
Perspective included health system	18*	86
Perspective included societal aspects	5*	24
Productivity costs were considered	3	14
Greenhouse gas emissions were considered	0	0
Study fully funded by industry	1	5
Study with only partial funding by industry	1	5
Disease/condition being primarily prevented or treated		
Cardiovascular disease	5	24
Cervical cancer	3	14
Obesity	2	10
Injuries	2	10
Sleep disorders	2	10
Other (all single disease/conditions)	7	33
Nature of the intervention		
Primary prevention (completely avert disease)	8	38
Secondary prevention/screening (slow/halt progression of disease)	6	29
Treatment/disease management	7	33
Includes universal interventions—i.e., whole population (even if just in scenario analyses)	4*	19
Includes targeted interventions— i.e., one particular population group (even if just in scenario analyses)	19*	90
Includes mandatory interventions (even if just in scenario analyses)	3*	14
Includes voluntary interventions (even if just in scenario analyses)	20*	95
Results (as per the key results in Table 2)		
Likely to be cost-saving	4	19
Likely to be cost-effective (ICER < NZ$ 45,000 per QALY/DALY/LY)	15	71
Not cost-effective	2	10

*For these characteristics, some studies included multiple categories, e.g., using both QALYs and LYs

**For the 11 studies not using a lifetime horizon the range was from 1 to 30 years, median = 2 years, mean = 9.6 years

The interventions studied were most commonly for primary prevention (38%), followed by treatment/management (33%) and then secondary prevention/screening (29%) (Table 2). Most of the interventions involved targeting a particular population group (90%) and were voluntary (95%), i.e., not involving regulations or taxes. Disease and condition topics were diverse, but 24% were related to cardiovascular disease and the next most common category was cervical cancer.

The search strategy also identified five CBAs (which did not have a cost-effectiveness component) and 12 other types of CEAs (albeit not using the QALY/DALY/LY metrics) (Table A3 in Additional file 1). Of the CBAs, only one included a cost-effectiveness component (i.e., cost-per-fall prevented [19]). Of the CEAs, only 17% (2/12) used a cost-per-death prevented, with the rest using other health metrics.

## Discussion

### Main findings and interpretation

It seemed possible to produce a reasonably coherent league table for the ICER values from different studies in this high-income country. However, given the method differences, such a league table is probably only useful as a general guide in terms of broad categorisations of “likely cost-saving”, “likely to be cost-effective”, and “unlikely to be cost-effective”. Indeed, relatively small differences between ICER results (e.g., less than $20,000 per QALY/DALY averted, or less than a twofold difference in ICER) may not be particularly critical to informed prioritisation of interventions (given the many other considerations needed in policymaking such as the size of the health gain, the size of the costs/cost-savings, impact on health inequalities, and intervention feasibility). However, differences larger than these, especially if premised on expert consideration of the assumptions and context of each study being compared, probably make the simple league table comparisons outlined in this study potentially useful for policymaking or at least guiding prioritisation around further research efforts.

The high levels of methodological diversity in the identified CEAs suggest the need for researchers to strive harder for methodological compatibility of their work, e.g., with the use of the ISPOR guidelines/CHEERS checklist [42] and other internationally agreed best practice health economic approaches [18]. This would allow their study results to be more readily integrated into methodologically compatible league tables. Similarly, if researchers perform CBAs, they should ideally also attempt to produce results in terms of cost per QALY gained from both a societal and health sector perspective.

The overall pattern of results of many cost-saving and cost-effective interventions was reasonably compatible with other league table work for this particular high-income country (the league tables in these three publications [11–13] and the online league table developed by BODE^3^ Programme [14]). They are also broadly compatible with Australian league table work [6, 7] and other international work on league tables—which has also identified both cost-saving and highly cost-effective interventions (see *Background*). This work collectively should therefore be considered by policymakers to guide research priorities and to inform at least the cost-effectiveness aspect of future intervention selection. Similarly, the league table work aimed at low and middle-income countries can sometimes identify interventions that could be considered for high-income countries like New Zealand (e.g., polypills for cardiovascular disease prevention, dietary salt reduction policies, and regulation around food advertisements) [4].

### Study strengths and limitations

A strength of this study is that it was the first attempt to produce a league table of interventions covering methodologically diverse health interventions for this particular high-income country. It also included relatively few studies that had industry funding (Table 2) which may reduce bias in the results [43]. Nevertheless, this work has a number of limitations as detailed below.

Firstly, the search strategy might have missed some published studies given that occasionally cost-effectiveness results might only be found in appendicised information and hence are not readily identified in literature searches. Publication bias may have resulted in some studies not appearing in the peer-reviewed literature if authors or journal editors/reviewers considered the results to be of relatively little interest to policymakers (e.g., perhaps for studies which reported very cost-ineffective interventions).

Secondly, there are limitations with the threshold we used for categorising cost-effectiveness (in terms of GDP per capita as per the WHO CHOICE approach [16]). Such an approach has been criticised in the literature (e.g., in several articles [44–46]), with a particular weakness being that it is not linked to the shadow price of a health system’s budget constraint. Nevertheless, its use here can be justified given that it provides some link to real-world resourcing capacity and because of the absence of agreed alternative options proposed for this particular case study country.

Thirdly, this study did not attempt to include the components of the size of the health gain and cost impacts in the league table because this is very challenging due to the need to allow for varying population sizes, discerning total population and target population focus, and standardising the time horizon. There was also still variation in the outcome measures (i.e., QALY/DALY/LY) and, of course, the size of these varies with the different discount rates used (hence differentially impacting the ICER results).

Fourthly, more sophisticated critiques of the identified studies are possible (e.g., applying the CHEERS checklist [42] or detailed comparisons with state-of-the-art guidelines for CEAs [18]). However, this was not justified given the results in Table 3 that already show substantive deviation from established guidelines for many of the studies (e.g., in the discount rate).

## Conclusions

It was possible to produce a reasonably coherent league table for the ICER values from different studies (within broad groupings) in this high-income country. Most interventions were cost-effective and a fifth were cost-saving. Nevertheless, study methodologies did vary widely and researchers need to pay more attention to using standardised methods that will allow their results to be included in future league tables.

## Additional file

Additional file 1: **Table A1.** Examples of the types of studies that were excluded from this review and league table development (albeit with some being briefly described in Table A3). **Table A2.** Additional commentary on the interventions in Table 2 of the main manuscript for the 21 New Zealand cost-effectiveness studies identified and published in the period 1 January 2010 to 8 October 2017 (ordered by decreasing cost-effectiveness, with additional details on each study in Table A4 and Table A5). **Table A3.** Additional cost-benefit analysis or other types of cost-effectiveness analyses identified for New Zealand in the period 1 January 2010 to 8 October 2017 (i.e., not using the QALY/DALY/LY metrics). **Table A4.** Summary of the methods characteristics of the 21 studies with cost-effectiveness analyses for New Zealand in the period 1 January 2010 to 8 October 2017 (included studies are ordered alphabetically by first author surname). **Table A5.** Results of the included studies for New Zealand in the period 1 January 2010 to 8 October 2017 (ordered alphabetically by first author surname). (DOCX 200 kb)

## Data Availability

All data used in this report are available on request to the authors upon reasonable request.

## Abbreviations

CBA: Cost-benefit analysis
CEA: Cost-effectiveness analysis
CUA: Cost-utility analysis
DALY: Disability-adjusted life-year
ICER: Incremental cost-effectiveness ratio;
LY: Life-year
NZ: New Zealand
QALY: Quality-adjusted life-year
