# Supplementary material for: Can cost-effectiveness results be combined into a coherent league table? Case study from one high-income country
Source: Popul Health Metr. 2019 Aug 5;17:10. doi: 10.1186/s12963-019-0192-x (PMC6683509; doi:10.1186/s12963-019-0192-x)
Supplement: Supplementary file 1 — Table A1. Examples of the types of studies that were excluded from this review and league table development (albeit with some being briefly described in Table A3). Table A2. Additional commentary on the interventions in Table 2 of the main manuscript for the 21 New Zealand cost-effectiveness studies identified and published in the period 1 January 2010 to 8 October 2017 (ordered by decreasing cost-effectiveness, with additional details on each study in Table A4 and Table A5). Table A3. Additional cost-benefit analysis or other types of cost-effectiveness analyses identified for New Zealand in the period 1 January 2010 to 8 October 2017 (i.e., not using the QALY/DALY/LY metrics). Table A4. Summary of the methods characteristics of the 21 studies with cost-effectiveness analyses for New Zealand in the period 1 January 2010 to 8 October 2017 (included studies are ordered alphabetically by first author surname). Table A5. Results of the included studies for New Zealand in the period 1 January 2010 to 8 October 2017 (ordered alphabetically by first author surname). (DOCX 200 kb) [file 12963_2019_192_MOESM1_ESM.docx]

**Additional file 1**

**Additional Methods**

Table A1: Examples of the types of studies that were excluded from this review and league table development (albeit with some being briefly described in Table A3)

| **Summary reason for exclusion** | **Reference/s for example study/studies** | **Reason for exclusion** |
| --- | --- | --- |
| A BODE^3^ Study | Nghiem et al 2016 [1] | This cost-utility analysis (CUA) of sodium reduction in NZ is an example of a study using the BODE^3^ Programme Protocol for CUAs (University of Otago, Wellington). We excluded such studies as these do use a standardised methodology and have already being included in an online interactive league table [2]. These cover dietary interventions (especially dietary sodium reduction), tobacco control, injury prevention, cancer prevention via HPV vaccination and screening, and other cancer treatment/management. |
| A cost-benefit analysis (CBA) (without an additional CEA) | Keall et al 2017 [3] | Takes a societal perspective and conducts a benefit-cost analysis (where the DALYs averted are monetised). (But a previous publication relating to this study did indeed conduct a CUA and is included in the results in Table 3, main manuscript). |
| Different metrics: cost per death prevented | Beliaev et al 2012 [4]; Wilson et al 2012 [5] | Only used cost per death prevented (i.e., did not use a QALY/DALY/LY metric). |
| Different metrics: cost per achieving a new health state | Elley et al 2011 [6]; Hayman et al 2017 [7] | Did not use a QALY/DALY/LY metric, e.g., for Elley et al the cost-effectiveness ratios were in terms of a person made ‘active’. For Hayman et al it was the cost per extra person vaccinated (also this was a CBA). |
| Cost-minimisation study | Chong et al 2014 [8]; Robertson et al 2015 [9]; Smart et al 2010 [10] | Typically these studies determined the cost-savings from a new intervention or altered management (but with no cost per integrated health metric calculated). |
| No intervention (e.g., cost of illness study) | Brown et al 2010 [11]; Costilla et al 2013 [12]; Wilson et al 2013 [13] | Considers QALYs or DALYs in terms of a disease burden/cost-of-illness study but no specified intervention. |
| Not published in the peer-reviewed journal literature | Health Technology Analysts 2010 [14] | A report which is not published in the peer-reviewed journal literature. |
| Outside the time period for this search | Hayes and Hansen 2007 [15] | Published prior to the search period (starting January 2010). |

**Additional Results**

Table A2: Additional commentary on the interventions in Table 2 of the main manuscript for the 21 New Zealand cost-effectiveness studies identified and published in the period 1 January 2010 to 8 October 2017 (ordered by decreasing cost-effectiveness, with additional details on each study in Table A4 and Table A5)

| **Study reference** | **Intervention*** | **Comments** |
| --- | --- | --- |
| ***Cost-saving interventions*** | |  |
| Leung et al 2012 [16] | Pedometer-based promotion in primary care versus time-based activity goals via green prescriptions | The 12-month timeframe for this study is a limitation, but even with on-going reductions in adherence it probably would remain cost-saving or cost-effective. |
| O’Keeffe et al 2012 [17]; And: Scott et al 2011 [18] | Diagnosis and treatment pathways for insomnia for a range of practitioners including, pharmacists, general practitioners (GPs), psychologists, other health professionals, and alternative health practitioners | There is some uncertainty about these results since some of the treatment cost data, on-referral rates, and treatment rates were self-reported by treatment providers and it is not known how representative this is. |
| Lew et al 2016 [19] | Primary HPV screening with partial genotyping in both unvaccinated women and cohorts offered vaccination | The study tested many strategies but with one (Strategy S2a involving 5-yearly HPV testing) being clearly the most cost-effective. |
| Friedman et al 2012 [20] | Proposed national programme to prevent paediatric abusive head trauma (AHT, often known as “shaken baby syndrome”) | There is likely to be very high uncertainty surrounding these results given the uncertainties around intervention effectiveness (though a trial to assess this [21] was underway at time of this publication by Friedman et al). The societal perspective taken includes consideration of health costs, but also costs associated with: community rehabilitation, special education, investigation and child protection, criminal trials, and punishment of offenders. |
| ***Cost-effective interventions*** | |  |
| Gander et al 2010 [22] | Diagnosis and treatment pathways for obstructive sleep apnoea syndrome (OSAS) from GP level through to surgical intervention | The cost time-frame was just one year which may not be entirely realistic. Furthermore, the lifetime QALY gain per person (of 5.4 years) was not discounted. Hence the ICER might be optimistic. |
| Lake et al 2013 [23] | Campylobacter control in NZ poultry meat supply: interventions at all points from farm to consumer (as per the situation in 2005) | There is high uncertainty around these results as they are based on disease epidemiology in 2005 (prior to a major decline in disease incidence following a regulatory intervention [24]) and potential other changes since then (as the industry considers export opportunities). Therefore, these interventions may no longer be cost-effective and this study may no longer be relevant to policy-makers. |
| Webb et al 2017 [25] | A “soft regulation” national policy for dietary sodium reduction that combines targeted industry agreements, government monitoring, and public education (modelled on the UK programme) | This study is conservative in that it did not consider health cost savings from the intervention. E.g., nearly all other sodium reduction interventions for NZ have been reported to be cost-saving [1] [26] (just one counselling intervention was not cost-saving but was still cost-effective [27]). |
| Maddison et al 2015 [28] | Improving exercise capacity and physical activity through a mobile phone / online intervention in addition to usual care, for people with ischaemic heart disease (IHD) | There is some uncertainty with these results given that physical activity was self-reported. Nevertheless, the primary outcome measure (change in peak oxygen uptake) was objectively measured. |
| Dalziel et al 2010 [29] | A broad range of interventions to prevent neural tube defects (from targeted promotion of folic acid supplement to voluntary/mandatory folic acid fortification of the food supply) | Other intervention results were not shown as these probably have very high uncertainty e.g., increasing folate levels in food. This is because of more recent information around dietary folate intake and increased cancer risk (e.g., the systematic review by Tio et al [30]). Even so there is also uncertainty around the impact of physician advice and health promotion campaigns. |
| Sopina and Ashton 2011 [31] | 18 different cervical cancer screening combinations (e.g., based on usage of the human papillomavirus (HPV) vaccine, screening interval length (3 or 5 years) etc) | There is some uncertainty around these results given that the model doesn’t account for herd immunity. Also this study may be less relevant to policy-makers given plans to introduce HPV testing to the cervical cancer control programme in NZ. |
| Panattoni et al 2012 [32] | Treatment of acute coronary syndrome with prasugrel if the person is a carrier of the CYP2C19*2 allele (if not a carrier of this allele, the person gets treatment with clopidogrel) | These results suggest cost-effectiveness. |
| Simms et al 2016 [33] | Strategies for screening for HPV in context of a nonavalent vaccine (“HPV9 vaccine”) | For 5 screens per lifetime, this was estimated to have a 100% probability of being cost-effective at a threshold of $42,000/LY saved. |
| Te Ao et al 2015 [34] | Increasing the use of thrombolysis treatment for ischaemic stroke by increasing hospital presentations and / or increasing use of thrombolysis treatment in hospital | The aim of this study was hypothetical i.e., to provide estimates of how much could be spent on hypothetical interventions to increase rates of using thrombolysis for stroke treatment in NZ hospitals, at cost-effective levels. |
| Te Ao et al 2012 [35] | Acute stroke units in NZ hospitals (as opposed to care on general wards) | A limitation was that data came from just one hospital and so these results may not be readily generalisable to other settings. |
| Keall et al 2015 [36] | Package of home modifications to reduce injuries from falls at home | Very likely to be cost-effective given the international literature relating to the home modification intervention and two modelling studies conducted in NZ (albeit just for the 65+ age-group [37] [38]). |
| Milne et al 2014 [39] | Long-term air humidification therapy plus usual care for people with moderate / severe COPD / bronchiectasis | Although based on a randomised controlled trial (RCT) this study had missing diary data and study withdrawals leaving only 87 patients in the analysis. Another limitation when considering long-term adherence is the relatively demanding nature of the intervention (involving 2 hours every day; a ‘cost’ not explicitly included in evaluation). |
| Rush et al 2014 [40] | A multicomponent through-school physical activity and nutrition programme (“Project Energize”) | There is large uncertainty around these results given that: (i) the comparison group was a historical cohort (albeit a limitation acknowledged by the authors); (ii) the long lag-times to health gains in adulthood, premised on an intervention effect assumed to decay at 1% per year after the first 5 years in the base-case – but the true decay rate is not known and could be very much higher. The authors noted that the model did not capture non-obesity related benefits that may be associated with increased physical activity (e.g., improved fitness, fundamental movement skills). But they did not discuss the mental health benefits of not being overweight or of the oral health benefits of improved nutrition. |
| Pinto et al 2013 [41] | Knee / hip osteoarthritis (OA) treatment: manual therapy, exercise therapy, or both, plus usual care | This study also included an additional analysis from a societal perspective which included productivity losses (giving more favourable results). |
| Carrasco et al 2011 [42] | Antiviral stockpiling for future influenza pandemics (relative to no stockpiling) | The major limitation is the high uncertainty around the effectiveness of antivirals as per recent reviews [43-46]. This study also included productivity costs, making it difficult to compare with other health system perspective evaluations. Results varied in scenario analyses but generally were cost-effective for stockpiles covering 10%+ of the population. |
| ***Not cost-effective interventions*** | |  |
| Harris et al 2011 [47] | Planned early start for dialysis treatment based on kidney function for patients with progressive chronic kidney disease. | This intervention would seem extremely unlikely to be cost-effective. The authors note that a limitation of the study was the dwindling response rates over time for the resource use log and the quality of life log. |
| Leung et al 2017 [48] | Exercise counselling intervention to enhance smoking cessation | A problem was the high non-adherence to the intervention (52% by the 7-day point). Targeting might improve this intervention’s cost-effectiveness but probably not enough to move it into the cost-effective category. |

Note: * The comparator is current practice/usual care unless otherwise specified (with more details in Table A4).

Table A3: Additional cost-benefit analysis or other types of cost-effectiveness analyses identified for New Zealand in the period 1 January 2010 to 8 October 2017 (i.e., not using the QALY/DALY/LY metrics)

| **Study reference** | **Intervention** | **Metric/s** |
| --- | --- | --- |
| Arnold et al 2016 [49] | Treatment for incontinence in women in rest home care | Measured reduced incontinence and costs (but didn’t specifically calculate cost per unit of reduced incontinence) |
| Beliaev et al 2012 [4] | Allogeneic red-blood-cell transfusion in severe symptomatic anaemia | Cost per death prevented |
| Boyd et al 2017 [50] | Border control intervention to stop the spread of pandemic influenza to NZ | CBA with QALYs monetised |
| Duncan 2014 [51] | Introduction of regulatory limits on contamination levels of *Campylobacter* in NZ | CBA and a net benefit reported |
| Elley et al 2011 [6] | Exercise on prescription with telephone support among women in general practice settings | Cost per level of increased activity |
| Foley et al 2011 [52] | Comparison of two modes of delivery of an exercise prescription scheme | Cost per person delivered the exercise prescription |
| Fyfe et al 2015 [53] | Community water fluoridation in NZ | Cost per population level of dental caries prevented |
| Hayman et al 2017 [7] | Supplementary measles immunisation in the highly immunised population of NZ | Cost per extra person vaccinated |
| Herd et al 2010 [54] | Provision of amethocaine (Ametop) compared with EMLA (eutectic mixture of local anaesthetics) for intravenous cannulation in a children's emergency department | Cost per case receiving topical anaesthesia |
| Keall et al 2013 [55] | Costs and benefits of a vehicle periodic inspection scheme with six-monthly inspections compared to annual inspections | Aspects of a CBA with an approximate threshold analysis |
| Keall et al 2017 [3] | Community level programme of home modifications | CBA where DALYs averted are monetised. Cost-effectiveness in terms of the cost per home fall injury prevented, was also calculated |
| Lee et al 2013 [56] | Primary prophylaxis with granulocyte colony-stimulating factor (GCSF) in patients with non-Hodgkin lymphoma (NHL) receiving CHOP (cyclophosphamide, vincristine, doxorubicin and prednisone) chemotherapy treatment | Cost of preventing one episode of febrile neutropenia |
| Lemanu et al 2013 [57] | Enhanced recovery versus standard care after laparoscopic sleeve gastrectomy | Cost per patient treated |
| Macmillan et al 2014 [58] | Specific policy changes e.g., to transform urban roads using best practice physical separation on main roads and bicycle-friendly speed reduction on local streets | CBA with benefit to cost ratios reported |
| McAuley et al 2010 [59] | A community-based obesity prevention program in children: the APPLE project | Cost per kg of weight gain prevented |
| Sammour et al 2010 [60] | Programme of “Enhanced Recovery After Surgery” after elective colonic surgery | Costs and health outcomes assessed but not integrated into a cost per improved outcomes |
| Wilson et al 2012 [5] | Provision of hospital care during an influenza pandemic | Cost per death prevented |

Table A4: Summary of the methods characteristics of the 21 studies with cost-effectiveness analyses for New Zealand in the period 1 January 2010 to 8 October 2017 (included studies are ordered alphabetically by first author surname)

| **Lead author/date and title of publication (reference)** | **URL link to abstract or article** | **Intervention: short** | **Intervention: longer** | **Comparator specification** | **Perspective** | **Time horizon (for costs and health gain accrued)** | **Population (including ethnicity, demographics, etc)** | **Metric (e.g., QALYs, DALYs or LYs)** | **Discount rate used (with note if different for costs and health gain)** |
| --- | --- | --- | --- | --- | --- | --- | --- | --- | --- |
| Carrasco et al 2011 [42] “Strategies for antiviral stockpiling for future influenza pandemics: a global epidemic-economic perspective” | <http://rsif.royalsocietypublishing.org/content/8/62/1307.long> | Antiviral stockpiling (influenza pandemics) | Antiviral stockpiling for future influenza pandemics | Presumed to be relative to no stockpile (though this is not specifically stated) | Societal (includes productivity costs) | 30 years | Populations for 10 countries. Included age-structure of NZ population. | QALYs | 10% |
| Dalziel et al 2010 [29] “Cost-effectiveness of mandatory folate fortification v. other options for the prevention of neural tube defects: results from Australia and New Zealand” | <https://www.ncbi.nlm.nih.gov/pubmed/19758481> | Folic acid / folate to reduce neural tube defects (NTDs) | Proposed interventions to reduce NTDs:  1. Promotion of folic acid supplement use one month before and three months after conception  2. Promotion of folate-rich food consumption  3. Extension (and maintenance) of voluntary folic acid fortification of the food supply  4. Mandatory folic acid fortification of wheat flour for bread-making. | Current practice / status quo.  The effectiveness data for this study were drawn from several international studies: each based on invention compared with the status quo in the particular study. | Health sector funder, based on Australian expenditure data (and converted to 2006 NZ$) | Lifetime for cost-utility analysis (up to age 80 years) | Population targets of interventions ranged from young women from minority groups, all women of childbearing age, to all healthy men and women in a particular area.  The effectiveness data from study was applied to the Australian and NZ populations. | DALYs | Costs and outcome were discounted at 5% per year (with 0% used in sensitivity analysis) |
| Friedman et al 2012 [20] “Primary prevention of pediatric abusive head trauma: a cost audit and cost-utility analysis | <https://www.ncbi.nlm.nih.gov/pubmed/23141137> | Paediatric Abusive Head Trauma prevention | Proposed national programme to prevent paediatric abusive head trauma (AHT, often known as “shaken baby syndrome”) | The study compared the costs and health outcomes for infants with AHT (that is, those who would be helped by the proposed intervention) to infants without AHT. | Societal perspective, direct costs only including:  hospital care, community rehabilitation, special education, investigation and child protection, criminal trials, punishment of offenders, lifetime-care for moderate or severe disability. | Lifetime | Costs were determined for a group of children aged under two years who had admitted to Starship Hospital anytime in 2004 to 2008, with an AHT diagnosis (mean age was 6.2 months, ethnicity breakdown was 62% Māori, 19% European, 13% Pacific, 6% other). The cost data were then applied to the NZ population. | QALYs | 3.5% per annum for costs and benefits |
| Gander et al 2010 [22] “Societal costs of obstructive sleep apnoea syndrome”. | <https://www.ncbi.nlm.nih.gov/pubmed/20927153> | Obstructive sleep apnoea treatment | Diagnosis and treatment pathways for obstructive sleep apnoea syndrome (OSAS) from GP level through to surgical intervention. | Untreated obstructive sleep apnoea syndrome. | Health sector funder for the CUA (study also has data on societal costs for the cost of illness part of the research – including productivity loss). | 12 months (2005 year) | NZers aged 30-60 years | QALYs | Not used (given the 12 month time frame) |
| Harris et al 2011 [47]; “Cost-effectiveness of initiating dialysis early: A randomised controlled trial.” | <https://www.ncbi.nlm.nih.gov/pubmed/21349618> | Starting dialysis treatment early. | Planned early start for dialysis treatment based on kidney function (Cockcroft-Gault estimated glomerular filtration rate of 10-14 mL/min/1.73m^2^), for patients with progressive chronic kidney disease. | Regular (“late”) start for dialysis treatment based on kidney function (Cockcroft-Gault estimated glomerular filtration rate of 5-7 mL/min/1.73m^2^), for patients with progressive chronic kidney disease. | Health sector (includes direct medical costs and patient transport costs etc). Study says “societal” but “health sector” more appropriate (e.g., income loss not included) | Study period was 9 years, with mean length of follow-up of 4.15 years | People with progressive chronic kidney disease (stage 5), from 32 renal units in Australia and NZ (642 people in total). | QALYs | 5% per year for costs and outcomes |
| Keall et al 2015 [36]; “Home modifications to reduce injuries from falls in the Home Injury Prevention Intervention (HIPI) study: a cluster-randomised controlled trial.” | <https://www.ncbi.nlm.nih.gov/pubmed/25255696> | Home modification fall prevention | Package of home modifications to reduce injuries from falls at home.  The intervention group had the package of home modifications made immediately. | No immediate home modification. The control group in the study had a three year wait for the package of home modifications. | Health sector funder | 20 years | People living in the Taranaki region, who owned their own home, where at least one occupant was a community services card holder, and the home was built prior to 1980 | DALYs | 3% per year |
| Lake et al 2013 [23]; “Cost-effectiveness or interventions to control campylobacter in the New Zealand Poultry Meat Food Supply.” | <https://www.ncbi.nlm.nih.gov/pubmed/23834790> | Campylobacter control in poultry | Campylobacter control in NZ poultry meat supply: interventions at all points from farm to consumer | Interventions compared with each other. | Societal: most costs were  NZ poultry industry perspective, benefits were reduction in disease from a NZ national perspective | 30 years | NZ population | DALYs | Costs were discounted at 3.5% per year; benefits were not discounted |
| Leung et al 2012 [16]; “Cost-effectiveness of pedometer-based versus time-based green prescriptions: the Healthy Steps Study.” | <https://www.ncbi.nlm.nih.gov/pubmed/23069363> | Pedometer promotion (physical activity) | Pedometer-based promotion in primary care versus time-based activity goals via green prescriptions | Time-based green prescriptions in primary care (standard green prescription delivery in NZ) | Health sector (includes out-of-pocket expenses) | 12 months | Relatively inactive community based adults aged 65+ years | QALYs (using EQ-5D) | Not used (given the 12 month time frame) |
| Leung et al 2017 [48]; “Economic evaluation of an exercise-counselling intervention to enhance smoking cessation outcomes: The Fit2Quit trial. | <https://www.ncbi.nlm.nih.gov/pubmed/28360828> | Exercise counselling for smokers | Usual Quitline support plus an exercise counselling intervention (up to ten face-to-face or telephone support sessions over six months) | Usual Quitline support (one-to-one telephone based support to quit smoking plus access to subsidised nicotine replacement therapy) | Health system | 24 week and lifetime | Adult Quitline users 2009-2012, detailed demographics provided in paper | QALYs | 3.5% per annum for costs and benefits (with scenario analysis for 0% and 5%) |
| Lew et al 2016 [19]; “Effectiveness modelling and economic evaluation of primary HPV screening for cervical cancer prevention in New Zealand.” | <https://www.ncbi.nlm.nih.gov/pubmed/27187495> | HPV screening | Primary HPV screening with partial genotyping in both unvaccinated women and cohorts offered vaccination | Current cytology-based screening programme (but for some ICERs the next best strategy was the comparator) | Health services (NZ Government) | Lifetime | NZ women aged 20–69 years | LYs and QALYs | 3.5% |
| Maddison et al 2015 [28]; “A mobile phone intervention increases physical activity in people with cardiovascular disease: Results from the HEART randomized controlled trial.” | <https://www.ncbi.nlm.nih.gov/pubmed/24817694> | Cellphone exercise intervention,  IHD | Improving exercise capacity and physical activity through a mobile phone / online intervention in addition to usual care, for people with ischaemic heart disease (IHD) | Usual care for IHD | Health sector funder | 24 week and 12 month | 24 week trial timeframe: Outpatients with clinically stable IHD from two Auckland hospitals, aged 18+ years; 81% male; 76% NZ European; mean age 60 years.  12 month scenario: applied to NZ population, conservative estimate of 2500 participants consenting nationwide | QALYs | Not used (given 12 month / 24 week timeframes) |
| Milne et al 2014 [39]; “Long-term air humidification therapy is cost-effective for patients with moderate or severe chronic obstructive pulmonary disease or bronchiectasis” | <https://www.ncbi.nlm.nih.gov/pubmed/24968990> | COPD/ Bronchiectasis humidification therapy | Long-term air humidification therapy plus usual care for people with moderate / severe COPD / bronchiectasis for 12 months | Usual care alone for people with moderate / severe COPD / bronchiectasis for 12 months | Health sector (includes out-of-pocket expenses) | 12 months | People diagnosed with moderate / severe COPD / Bronchiectasis | QALYs (based on scores from the St George’s Respiratory Questionnaire – SGRQ) | 3.5% for costs only (calculated for a five year period then annualised to fit the 12 month time horizon) |
| O’Keeffe et al 2012 ([17]; “Insomnia treatment in New Zealand.”  And: Scott et al 2011 [18]; “Insomnia – treatment pathways, costs and quality of life.” | <https://www.ncbi.nlm.nih.gov/pubmed/22327158>  and:  <https://www.ncbi.nlm.nih.gov/pubmed/?term=21693060> | Insomnia treatment | Diagnosis and treatment pathways for insomnia for a range of practitioners including, pharmacists, GPs, psychologists, other health professionals, and alternative health practitioners. | Untreated insomnia | Health sector (since most costs were medical costs, with the non-medical costs being transport for treatment costs). | 12 months | NZers aged 20-59 years | QALYs | Not used (given the 12 month time frame) |
| Panattoni et al 2012 [32]; “The cost effectiveness of genetic testing for CYP2C19 variants to guide thienopyridine treatment in patients with acute coronary syndromes: A New Zealand evaluation”. | <https://www.ncbi.nlm.nih.gov/pubmed/22974536> | Acute coronary syndrome (ACS) treatment | Treatment of acute coronary syndrome with prasugrel if the person is a carrier of the CYP2C19*2 allele. | Treatment of ACS with generic clopidogrel (if not a carrier of the allele). | Health sector funder | Lifetime | NZers aged 45-80 years hospitalised with an ACS | QALYs | 3% per year, costs and benefits |
| Pinto et al 2013 [41]; “Manual therapy, exercise therapy, or both, in addition to usual care, for osteoarthritis of the hip or knee. 2: economic evaluation alongside a randomised controlled trial” | <https://www.ncbi.nlm.nih.gov/pubmed/23811491> | Knee / hip osteoarthritis treatment | Knee / hip osteoarthritis (OA) treatment: manual therapy, exercise therapy, or both, plus usual care. | Usual care for knee / hip OA | Presented for two perspectives: NZ health system perspective, and societal perspective (including productivity loss) | 12 months | NZ patients with hip / knee osteoarthritis attending GPs / referred to hospital orthopaedic outpatient unit; 98% NZ European, 55% female, mean age 66 years. | QALYs | Not used (given the 12 month time frame) |
| Rush et al 2014 [40]; “Lifetime cost effectiveness of a through-school nutrition and physical programme: Project Energize” | <https://www.ncbi.nlm.nih.gov/pubmed/24743006> | Obesity prevention in schools | A multicomponent through-school physical activity and nutrition programme (“Project Energize”) | School children in non-intervention schools in the same region of NZ | Health sector funder (excluding out-of-pocket expenses) | Lifetime (to age at death or age 100y) | Primary school children aged 6-8 and 9-11 years (but delivered to their teachers also). Sex, ethnicity & school SES studied. | QALYs | 3.5% (both) |
| Simms et al 2016 [33] “Will cervical screening remain cost-effective in women offered the next generation nonavalent HPV vaccine? Results for four developed countries.” | <https://www.ncbi.nlm.nih.gov/pubmed/27541596> | Screening for HPV | Strategies for screening for HPV in context of a nonavalent vaccine (“HPV9 vaccine”) | Two benchmarks: current practice (cytology based screening) and for cohorts offered HPV vaccination | Health-services | Lifetime | Women aged 25–74 (for screening) | LYs | 3% |
| Sopina and Ashton 2011 [31]; “Cost-effectiveness of a cervical screening program with human papillomavirus vaccine”. | <https://www.ncbi.nlm.nih.gov/pubmed/21936972> | HPV vaccine, cervical screening | 18 different intervention combinations based on usage of the human papillomavirus (HPV) vaccine, age at cervical screening start (20, 25, or 30 years), screening interval length (3 or 5 years), age at screening cessation (60, 65, or 69 years). | No vaccine and usual cervical cancer screening (women aged 20-69 years, every three years). | Health sector funder | Lifetime (followed girls from age 12 to 85 years or death) | NZ girls and women aged 20-85 years | LYs and QALYs | Cost and QALYs discounted at 3%.  Also examined effect of varying discounted rates, as part of sensitivity analysis. |
| Te Ao et al 2012 [35]; “Are stroke units cost effective? Evidence from a New Zealand stroke incidence and population-based study.” | <https://www.ncbi.nlm.nih.gov/pubmed/22010968> | Acute stroke units | Acute stroke units in NZ hospitals | Stroke care on a general hospital ward. | Health sector funder (direct medical costs) | 12 month and lifetime | Stroke sufferers admitted to Middlemore Hospital in Auckland 2002 – 2003. Females made up 53%. Māori 11%, Pacific 19%. Average age was 70.3 years. | QALYs | 3% per year for costs and gains beyond 1 year. |
| Te Ao et al 2015 [34]; “Potential gains and costs from increasing access to thrombolysis for acute ischemic stroke patients in New Zealand hospitals.” | <https://www.ncbi.nlm.nih.gov/pubmed/?term=24206567> | Increased thrombolysis stroke treatment | Increasing the use of thrombolysis treatment for ischaemic stroke by increasing hospital presentations and / or increasing use of thrombolysis treatment in hospital | Current levels of thrombolysis treatment amongst stroke patients | Health sector funder | 12 month and lifetime | An estimated 6000 stroke sufferers per year, in NZ. | QALYs | Not stated. |
| Webb et al 2017 [25] “Cost effectiveness of a government supported policy strategy to decrease sodium intake: global analysis across 183 nations.” | <https://www.ncbi.nlm.nih.gov/pmc/articles/PMC5225236/> | Dietary sodium reduction | A “soft regulation” national policy that combines targeted industry agreements, government monitoring, and public education (modelled on the UK programme) | Comparative risk assessment approach – so the business-as-usual state | Not stated but likely to be a health system perspective | 10 years | Populations of 183 countries, also by age, race (in African countries) and hypertensive status | DALYs | 3% |

Table A5: Results of the included studies for New Zealand in the period 1 January 2010 to 8 October 2017 (ordered alphabetically by first author surname)

| **Lead author/date** | **Result: health gain** | **Result: net costs (year of costs)** | **Result: Intervention cost only (e.g., programme delivery costs)** | **Result: Changes in health care costs included in net costs (Yes/No)** | **Result: ICER (year of $NZ)** | **Heterogeneity alert** | **Study funder** | **Comments on any key aspects, including limitations** |
| --- | --- | --- | --- | --- | --- | --- | --- | --- |
| Carrasco et al 2011 [42] | Not reported (but graphs show changes in fatality rates by stockpile levels) | Approximately US$ 0.9 billion (includes productivity costs) (costing year not stated, presumably 2010) | No total cost presented but components in Supplementary Material e.g., $US 20 per antiviral course for NZ | Yes | Approximately US$20,000 per QALY (actually between (US$15,000 and 20,000 per QALY for stockpile sizes covering 10-30% of the population (Fig S3.6 i.e., of 30% of misallocation and misdiagnosis of antivirals) | Results for 10 countries – but nil for in-country heterogeneity. | National University  of Singapore | The major limitation is the high uncertainty around the effectiveness of antivirals as per recent reviews.[43] [44] [45] [46] This study also included productivity costs. Scenario analysis of a vaccine delay (240 to 350 days) also indicated that stockpiling was generally cost-effective for stockpiles covering 10%+ of the population. Also under US$5000 per QALY for NZ in the case of generic antivirals not protected by a patent (albeit only at a price of $0.02 per course). |
| Dalziel et al 2010 [29] | Based on literature. Discounted reduction in DALY ranges from 39 (dietary folate, targeted) to 722 (Health promotion, supplement use) for NZ | Discounted net costs, modelled to life expectancy, ranged from $-602 (voluntary fortification) to $79.5 million (mandatory fortification, bread) (2006 NZ$) | Ranged from NZ$7307 (voluntary fortification) per year to NZ$2.97 million (mandatory fortification, bread) per year | Yes, the health system costs averted due to reduction in NTD numbers were included in net costs | The cost per DALY averted ranged from cost savings (voluntary fortification) to NZ$148,700 (dietary folate, targeted campaign). Folic acid supplementation was a cost-effective option (NZ$2700 – NZ$6500 for different delivery mechanisms). Mandatory fortification was not found to be cost effective for NZ. | Some of the included interventions were designed to target specific sub-populations (minority ethnic groups, younger women). | Food Standards Australia and NZ (a bi-national government agency) | The study was an assessment of possible interventions (intervention health gain estimated based on the literature).  Limitations of the research included:  Not all possible intervention options could be included in the study.  Interaction effects between interventions were not able to be assessed.  Some of these results probably have very high uncertainty e.g., increasing folate levels in food. This is because of more recent information around dietary folate intake and increased cancer risk (e.g., the systematic review by Tio et al 2014[30]). |
| Friedman et al 2012 [20] | Based on literature for utility loss of adult head trauma, ranges from 0.12 (least severe case) to 1.0 (fatal case) per case averted | Estimated for different combinations of intervention effectiveness (reduction in AHT) and programme cost (2012 NZ$) | Based on literature. Estimated as ranging from $20 to $100 per new-born | Yes, detailed health and other costs provided for cases of AHT (which would be avoided with proposed programme) for range of severities. | Cost-saving where reduction in AHT is 30% or more and intervention cost is between $20 and $100 per new-born. While most estimates were cost-saving, some estimates were as high as 471,000 per QALY | Analysis provided was for whole NZ, no further gender or ethnic breakdown. | Funded by the Geddes Foundation, through the Starship Foundation (children’s hospital) | The study was an assessment of a possible intervention (intervention health gain estimated based on literature).  Study strength was the detailed breakdown of direct societal costs (e.g., including education) determined through the review of 52 cases of AHT.  Limitations: QALY values for lost utility were based on adult cases of AHT from literature, “savings” from reduction in AHT rate may not change expenditure levels but would free up money to be used for additional work, questions around intervention effectiveness (large trial to assess this underway at time of publication). |
| Gander et al 2010 [22] | Based on literature. Estimated as 5.4 gain in QALYs per treated case, with around 20% of cases treated = total gain of 102,531 QALYs per year | These are presented as the direct medical costs per cases treated ($649.63) – direct medical costs per untreated case ($142.84) = $506.79 per QALY per year. (2005 NZ$) | The study presents the healthcare costs of treatment and diagnosis as the intervention cost. | The study presents the changes in healthcare costs as the net costs. | Direct medical cost per QALY gained was estimated to be $94. | Conducted for the NZ population aged 30 – 60 years as a whole (no further gender or ethnic breakdown) but noted that the simulation tool (the outcome tree and decision model) could be used to assess economic effect of OSAS treatment for different population groups (especially Māori). | Massey University Wellington Strategic Research Fund | The study assessed the effect of possible intervention to diagnose and treat OSAS (intervention health gain estimated based on literature).  Limitations of the study include:  Over-estimates in costing could have resulted as literature on comorbid OSAS risks are based on severe OSAS cases;  The outcome tree was based on practice in Wellington, which may not be the same in the rest of NZ.  To accurately estimate the ICER it may have been desirable to discount the lifetime QALY gain (5.4 years) and to consider more than just one year of intervention costs. |
| Harris et al 2011 [47] | No sig diff between groups (early group: 1.97 QALYs per person, late group 2.07 QALYs per person, after discounting) | Mean costs per patient (2008 AU $) of study duration were $18,715 higher in the early start group than the late start group ($215,354 early group, $202,124 late group) but not sig different. | Mean dialysis costs and costs for transport for dialysis per person were sig. higher in the early start group:  Early start dialysis 2008 AU $117,163 versus late $96,763  Early start transport for dialysis $4459 versus late start $4132. | Yes (in addition to the dialysis costs which were the main part of the intervention costs) | 72% of results indicated reduced health gain and increased costs. Only 0.3% of iterations gave a positive QALY at < $50,000 per QALY | No extra analysis presented for sub-groups (not even by Australia versus NZ). | National Health and Medical Research Council of Australia, Australian Health Ministers Advisory Council, Royal Australian College of Physicians / Australian and NZ Society of Nephrology, National Health Foundation (NZ and Australia), Baxter Healthcare Corp, Health Funding Authority NZ, International Society for Peritoneal Dialysis, Amgen Australia Ltd, Janssen Cilag Pty Ltd. | This study measured utility directly (rather than using values from literature).  The study was a randomised controlled trial where patients were randomly selected for early or late start dialysis groups.  The authors note that a limitation of the study was the dwindling response rates over time for the resource use log and the quality of life log – though risks for the study around this were mitigated by the fact that response rates remained similar over time in the early and late start groups.  As there is no information in the paper about how NZ costs compare to the Australian costs, we can only assume that the findings reported in the paper generalise to NZ on its own (though it is possible that they may not). |
| Keall et al 2015 [36] | 15,800 DALYs prevented over 20 years. | See intervention costs. | The home modifications cost $564 on average per dwelling upfront (2012 NZ$) Applied over the whole country, this would be almost $980 million as a one-off cost. | No, changes in healthcare costs were not reported. | The cost of the intervention per discounted DALY prevented was $14,300. | No extra cost analysis presented for sub-groups – though did note that the HIPI study has been extended to investigate the effect of home modifications for Māori population specifically. | Health Research Council of NZ | This was a cluster randomised controlled trial (actual intervention implemented and real health gains measured), which had a cost-utility analysis component. Limitations of the study included: Unknown matching rates for administrative injury data to study participants based on name, address, date of birth. Generalisability: the intervention was carried out on houses which have low rates of maintenance – not all dwellings will be in this shape. Had to exclude people who moved out of their modified homes or died from the study. The sample size was not large enough to conduct sub-analyses. |
| Lake et al 2013 [23] | Reduction in DALY per year ranged from 8 (consumer hygiene education) to 1010 (Irradiation at the primary processing stage) | Net costs included only the intervention costs and ranged from NZ$0.16 million (consumer education) to NZ$43.9 million (irradiation) (2009 NZ$) | See net costs | No, changes in healthcare costs were not included. | Cost per DALY averted ranged from NZ$1200 (primary processing interventions) to NZ$43,400 (irradiation at primary processing stage) | No extra analysis presented for NZ population sub-groups | Research partly based on student honours project; Ministry for Primary Industries funded the development of the poultry food chain model used in the study. | The study assessed the effect of possible interventions to reduce campylobacter in the NZ food supply (where the intervention health gain was estimated based on literature, which is acknowledged as a limitation in the paper). Assessed effect of interventions based on 2005 epidemiology (prior to any actual implementation of interventions).  Didn’t include any estimates of effect of interventions in terms of healthcare costs. |
| Leung et al 2012 [16] | Marginal utility gain per person of 0.035 (0.044 – 0.009) | -$79 per person ($1722 - $1801); (Year=2008) | Around $50 per person (for the pedometer) | Yes | Cost saving (-4999 per QALY gained) | Nil (no extra analyses by sex/ethnicity/disability within the 65+ age-group) | HRC and Sports and Recreation NZ | This was an evaluative study (actual intervention implemented and real health gains measured), based on a RCT conducted in 2006 and 2007. Included out-of-pocket costs. Also calculated ICERs per 30 minutes of moderate intensity physical activity. A limitation is the 12-month timeframe as there is the possibility that pedometer use would not be maintained in the long-term. The lead author is a current member of the BODE^3^ Team but this work was conducted previously. The cost results may be somewhat out-dated given pedometers are now free with modern smartphones. |
| Leung et al 2017 [48] | 24 week: intervention group gained 0.001 QALYs per person but was not significant.  Lifetime:  Adherent intervention group gained 0.057 (male) and 0.068 (female) QALYs per person between the ages of 40 and 100 yrs. | 24 week:  Programme costs only  Lifetime:  Mean costs per person for males were:  $9952 (intervention) and $9700 (usual care)  Mean costs per person for females were:  $11,032 (intervention group)  and $10,833 (usual care) | Over 24 weeks: Intervention cost was $428 (2012 NZ$) per person, based on intervention group costs (Exercise plus usual Quitline) of $623 per person minus Quitline alone cost of $195 per person. | 24 week:  No  Lifetime:  Yes | 24 week:  NZ$451,000 per QALY gained  Lifetime: (for adherent participants)  NZ$4431 (male) and NZ$2909 (female) | Separate lifetime analysis for males and females. | Health Research Council of NZ and the Heart Foundation. | Strengths: was a large (906 participants) randomised controlled trial with conservative costings.  Limitations: quit status not verified; Markov model used for lifetime study only had CVD and lung cancer – didn’t include other cancers / diseases; Markov model risk parameters for lung cancer were for heavy smokers (may not be appropriate to user for lighter smokers); the lifetime analysis was conducted for “adherent” intervention participants (that is people with 7 or more intervention contacts) versus usual care – non-adherent intervention participants weren’t included in the lifetime analysis. |
| Lew et al 2016 [19] | Not clear (missing footnotes in the Supplementary Material) | For strategy S2a (unvaccinated scenario): -$1.3 million compared to current practice (but still $30.4 million) (2015 NZ$) | Not distinguished from net costs | Yes | Cost-saving relative to current practice; One strategy (S2a): $20,602 per QALY saved in unvaccinated scenario; $9,769 in vaccinated scenario (both compared to next best strategy) | Considered vaccinated and non-vaccinated groups | Ministry of  Health, NZ | The study tested many strategies but with one (Strategy S2a involving 5-yearly HPV testing) being clearly the most cost-effective (albeit more so for the unvaccinated population). The study did not consider impacts for Māori women separately. |
| Maddison et al 2015 [28] | No significant difference between intervention and comparator on EQ-5D results | Only intervention setup and delivery costs were detailed in the paper | Set up and delivery costs for HEART intervention during trial (24 week period) were $20,313 (2012 NZ$ excluding GST) or $239 per participant | No | For the 24 week trial: $28,768 per QALY  Applied to NZ population, 12 month timeframe:  $2693 per QALY | No separate analysis for specific age, sex, ethnic groups. Did note that the intervention could easily be tailored to specific demographic groups. | Health Research Council of NZ and the Heart Foundation. | Study strengths include: primary outcome measure objectively (change in peak oxygen uptake), and used computer allocation to groups so study was single-blind and groups were balanced for baseline factors.  Limitations: secondary outcome (physical activity) was self-report (potential for recall bias). |
| Milne et al 2014 [39] | Mean gain of 0.068 QALYs  per person per year | $1417 (Table 4) (2012-2013 NZ$) | $2059 per person per year | Yes,– the intervention group healthcare costs were $642 less than the control healthcare costs (excludes intervention programme costs) | Mean cost per QALY was NZ $20,902 | The analysis was presented for a specific group defined by their health condition (COPD / bronchiectasis) and severity (mod / severe). Despite the narrow definition, there could well be differences within this group for different ethnic and SES groups but this was not explored. | Fisher & Paykel Healthcare  (manufacturer of the humidification device). | This was an evaluative study (actual intervention implemented and real health gains measured). Based on a 12 month RCT conducted with 108 patients. However, due to missing diary data and study withdrawals only 87 patients were included in this analysis (making study size a limitation). Additional limitations include the lack of blinding in the study (patients knew they were using the humidification device) and the derivation of utility scores from SGRQ answers. A key healthcare cost driver was hospital discharges. These costs were estimated using cost weights and unit cost – reflecting the average price paid across NZ rather than the specific costs associated with these particular hospital discharges – another area of potential inaccuracy. It was also noted that adherence to the therapy (involving 2 hours every day) makes this a demanding intervention. |
| O’Keeffe et al 2012 [17]; And: Scott et al 2011 [18] | Based on literature. Used 0.157 QALYs gain per person treated (per year) | Net costs for treating someone with insomnia estimated to be -$482 per person per year (a saving). Based on $145 to treat insomnia - $628 for untreated insomnia* | The study presents the costs of treatment and diagnosis as the intervention cost (average of $145 per person). | The study presents the changes in healthcare costs as the net costs. Only costs associated with insomnia are included (no comorb condition costs). | The cost per QALY gained was estimated to be -$3072 (a saving). | There were no additional breakdowns, despite literature indicating differences in insomnia rates by ethnicity, age, and SES. | NZ Lottery Health Grants Board | The cost utility analysis in the Scott et al (2011) paper presented the same data as the O’Keeffe et al (2012) study. O’Keeffe et al present more information about insomnia treatment options, including statistics on success rates, number of consults, types of treatment offered by practitioner etc. The O’Keeffe et al study had limited information about how the cost for untreated insomnia is calculated; more detail about this cost calculation is provided in the Scott et al (2011) paper. The studies both assessed the effect of possible intervention to diagnose and treat insomnia (where intervention health gain was estimated based on literature). One limitation noted by the authors was that some of the treatment cost data, on-referral rates, and treatment rates were self-reported by treatment providers and it is not known how representative this is. |
| Panattoni et al 2012 [32] | Mean patient lifetime QALYs gained ranged from 8.057 (prasugrel only) to 8.476 (genetically guided prasugrel / clopidogrel use) compared to 8.411 for clopidogrel only; all based on national hospital data. Health gain for four different ethnic groups given in paper. | Based on national hospital data, mean patient lifetime total costs (2009 NZ $):  $84,298  Clopidogrel only,  $82,717  Prasugrel only,  $84,863  Genetically guided prasugrel / clopidogrel use. | Based on national hospital data, mean patient costs (2009 NZ $) for medication (and genetic test for the genetically guided strategy):  $161  Clopidogrel only,  $758  Prasugrel only,  $444  Genetically guided prasugrel / clopidogrel use. | Yes. | Based on national hospital data, (2009 NZ $):  Comparing clopidogrel only (A) to prasugrel only (B) the IER is $4476 per QALY gained (B minus A)  Comparing clopidogrel only (A) to genetically guided use of prasugrel / clopidogrel (C) the ICER is $8702 per QALY gained  (C minus A) | Analysis presented for four different ethnic groups: NZ European, Māori, Pacific, and Asian, with costs, QALYs and ICER for each.  For the ICER based on national hospital data, the genetically guided use of prasugrel / clopidogrel was especially cost effective for Māori ($7312/QALY)and Pacific peoples ($7041/QALY) | Auckland District Health Board A-Plus Trust (part of the NZ public health system). | Intervention health gain based on literature.  The study also provided a comparison of two different sources of adverse events rates (those derived from the TRITON-TIMI 38 study and those derived from national hospitalisation data), and two different economic analyses were presented based on these rates, as part of a sensitivity analysis. Some differences between the two sets of rates are noted in the paper (e.g. inclusion or conditions / disease coding differences), which is a possible limitation in terms of how comparable the two analyses are. |
| Pinto et al 2013 [41] | All 3 therapies (Tx) produced increases in QALYs per year compared to usual care. Greatest was for Exercise therapy (0.687 QALYs per person per year) | Total health system costs ranged from $3931 (usual care, mean costs per person per year) to $5574 (combined Tx). Societal costs were ranged from $6131 (manual therapy to $7565 (combined Tx). | Mean annual costs per person:  Manual therapy $486, Exercise therapy $503, Combined therapies $507 | Yes, changes in healthcare costs are included in net costs | Mean cost per QALY gained:  Health system perspective ranged from $26,400 (exercise Tx) to $148,639 (combined Tx)  Societal perspective ranged from $-38,072 (manual Tx) to $53,216 (combined Tx) | No breakdown by age, sex, ethnic group. Very high proportion of participants were NZ European (98%) | Health Research Council of NZ and NZ Lottery Health Grants Board | All costs in 2009 NZ $.  Strengths of study:  Economic evaluation was based on RCT of the three treatment options.  Included a sub-analysis where people who had joint replacement were excluded. |
| Rush et al 2014 [40] | 0.007 (younger) and 0.009 (older) QALYs per capita (each age-group),  Māori figures for the same age groups were 0.008 (young) and 0.010 (older) QALYs. | Extra $217 and $219 per capita over lifetime (each age-group) (year=2011) | NZ$1,891,175 in 2010; $44.96/child/year | Yes | “$30,438 [per QALY] for the younger and $24,690 for the older children, and lower for Māori (younger $28,241, older $22,151)” | Yes: different ages, Māori, and school SES | Ministry of Health assisted in funding the evaluation of the intervention | This was an evaluative study (actual intervention implemented and real health gains measured). The main obesity measure in the study involved the comparison of BMI for younger and older children from the intervention group (2011 data) with BMI for historical cohorts from the same NZ region (from 2006 for the younger group and from 2004 for the older group). This historical comparison is noted as a limitation, in that the study authors assume that there were no changes (aside from the programme) between the time periods. The intervention effect was assumed to decay at 1% per year after the first 5 years in the base-case – but this decay rate is not known and could be much higher (and even higher than the 10% pa decay in a sensitivity analysis). The results are dependent on the lifetime epidemiology and costing model in a Report by Health Technology Analysts (available online). The article includes a league table of obesity prevention interventions, but the source is not clearly documented. |
| Simms et al 2016 [33] | Not clear (see Comments) | Not clear (2013 NZ$) | Not clear for the programme (but some specific per individual costs are in Appendix 1) | Yes for cervical cancer | $5000 per LY saved for 5 screens per lifetime (for cohorts offered nonavalent vaccine) | Nil | NHMRC (Australia) | For 5 screens per lifetime, estimated to have a 100% probability of being cost-effective at a threshold of $42,000/LY saved. Some data were not clear (e.g., the “discounted life years” in Fig 3a). This was not clarified in the Appendices either. It was not clear why other countries had a cost per GP visit but not NZ (Appendix 1). Given the focus on screening – the cost of the nonavalent vaccine was not considered. |
| Sopina and Ashton 2011 [31] | Total population QALYs gained ranged from 58.7 to 58.9. | Total costs ranged from 2009 NZ $3603 to $11,016. | Different combinations of total intervention cost not given but component costs used in model were supplied – e.g. 2009 NZ $450 for three doses of HPV vaccine Gardasil. | Yes, costs include cervical cancer screening and treatment costs | ICER with “no vaccine” base case comparison ranged from 2009 NZ $3562 to $10,169 per QALY gained. | The model trialled different screening start and end ages. No analysis by ethnicity. | No external funder described. Researchers from University of Auckland. | There is some uncertainty around these results given that the model doesn’t account for herd immunity. QALY rates were based on literature. In addition, if NZ HPV incidence rates are higher than the estimated ones used (which were not based on NZ data) interventions in the study may be more cost-effective. |
| Te Ao et al 2012 [35] | The intervention produced a 0.08 gain in QALYs in the first year (Intervention 0.53 – comparison 0.44) and 1.52 QALYs over lifetime. | Stroke unit costs were $24,275 (first year) and $77,313 (lifetime) compared to $20,849 (first year) and $67,057 (lifetime) for the general ward (2008 NZ$) | The key intervention cost was $1100 per person per day for a stroke unit admission compared to $850 per person per day for an admission to the general ward. | Yes. | The ICER (stroke unit – general ward) was $42,813 per QALY (first year) and $6747 per QALY (lifetime). | No breakdown by age, sex, ethnic group. | No external funding source. | Intervention health gain estimated based on literature.  Limitation:  Based on stroke unit and general ward operations in one hospital – may not generalise to other settings. |
| Te Ao et al 2015 [34] | Per person QALYs in first year: 0.54 with thromb. Tx and 0.48  QALYs without (lifetime):  4.79 with thromb tx, 4.18 without. | $1621 per person in the first year, $4051 per person over lifetime. (2010 NZ$) | Cost of thrombolysis estimated to be NZ$2043 per person | Yes. | In the first year: $27,017 per QALY;  Lifetime: $6641 per QALY | No analysis presented for different population sub-groups | NZ Stroke Foundation | The main aim of study was to provide estimates of how much could be spent on hypothetical interventions to increase rates of thrombolysis use for stroke treatment in NZ hospitals, at cost-effective levels.  The intervention gains were derived from literature. |
| Webb et al 2017 [25] | 9639 (NZ only); 3.4 DALYs per 1000 adults (NZ only) | Estimated at 14 million per year for NZ (for a 10 year intervention) (assuming 2013 I$ as this is the year for epidemiology data) | I$3.36 per capita to implement (of which around I$1.26 is for human resources and I$1.07 for mass media campaigns) | No | I$989/DALY (for NZ) | Results for 183 countries; but not for different groups within countries | National Heart, Lung,  and Blood Institute | Results are shown separately for NZ in the Supplementary Material. Did not consider health cost savings from the intervention – so a conservative analysis. |

**References**

1. Nghiem N, Blakely T, Cobiac LJ, Cleghorn CL, Wilson N. The health gains and cost savings of dietary salt reduction interventions, with equity and age distributional aspects. BMC Public Health*.* 2016;16(1):423.

2. University of Otago & University of Melbourne. ANZ-HILT: Australia and New Zealand Health Intervention League Table (Vers 2.0) 2019 [Available from: <https://league-table.shinyapps.io/bode3/>].

3. Keall MD, Pierse N, Howden-Chapman P, Guria J, Cunningham CW, Baker MG. Cost-benefit analysis of fall injuries prevented by a programme of home modifications: a cluster randomised controlled trial. Inj Prev*.* 2017;23:22-26.

4. Beliaev AM, Marshall RJ, Gordon M, Smith W, Windsor JA. Clinical benefits and cost-effectiveness of allogeneic red-blood-cell transfusion in severe symptomatic anaemia. Vox Sang*.* 2012;103(1):18-24.

5. Wilson N, Nhung N, Higgins A, Kvizhinadze G, Baker MG, Blakely T. A national estimate of the hospitalisation costs for the influenza (H1N1) pandemic in 2009. N Z Med J*.* 2012;125(1365):16-20.

6. Elley CR, Garrett S, Rose SB, O'Dea D, Lawton BA, Moyes SA, Dowell AC. Cost-effectiveness of exercise on prescription with telephone support among women in general practice over 2 years. Br J Sports Med*.* 2011;45(15):1223-1229.

7. Hayman DTS, Marshall JC, French NP, Carpenter TE, Roberts MG, Kiedrzynski T. Cost-benefit analyses of supplementary measles immunisation in the highly immunized population of New Zealand. Vaccine*.* 2017;35(37):4913-4922.

8. Chong V, Zhou L, Hundal H, Koea J. Acute surgical treatment of cutaneous abscesses: cost savings from prioritisation in theatre. N Z Med J*.* 2014;127(1399):51-57.

9. Robertson J, Linkhorn H, Vather R, Jaung R, Bissett IP. Cost analysis of early versus delayed loop ileostomy closure: a case-matched study. Dig Surg*.* 2015;32(3):166-172.

10. Smart OC, Sykes P, Macnab H, Jennings L. Testing for high risk human papilloma virus in the initial follow-up of women treated for high-grade squamous intraepithelial lesions. Aust N Z J Obstet Gynaecol*.* 2010;50(2):164-167.

11. Brown P, McNeill R, Leung W, Radwan E, Willingale J. Current and future economic burden of osteoporosis in New Zealand. Appl Health Econ Health Policy*.* 2011;9(2):111-123.

12. Costilla R, Tobias M, Blakely T. The burden of cancer in New Zealand: a comparison of incidence and DALY metrics and its relevance for ethnic disparities. Aust N Z J Public Health*.* 2013;37(3):218-225.

13. Wilson R, Derrett S, Hansen P, Langley JD. Costs of injury in New Zealand: Accident Compensation Corporation spending, personal spending and quality-adjusted life years lost. Inj Prev*.* 2013;19(2):124-129.

14. Health Technology Analysts. Cost effectiveness report of public health interventions to prevent obesity. Report prepared for the Heatlh Research Council of New Zealand. Sydney: Health Technology Analysts Pty Ltd; 2010. <http://www.victoria.ac.nz/sog/researchcentres/health-services-research-centre/docs/downloads/CE-Obesity-Prevention-Full-Report-publish.pdf>

15. Hayes JL, Hansen P. Is laparoscopic colectomy for cancer cost-effective relative to open colectomy? ANZ J Surg*.* 2007;77(9):782-786.

16. Leung W, Ashton T, Kolt GS, Schofield GM, Garrett N, Kerse N, Patel A. Cost-effectiveness of pedometer-based versus time-based Green Prescriptions: the Healthy Steps Study. Aust J Primary Health*.* 2012;18(3):204-211.

17. O'Keeffe KM, Gander PH, Scott WG, Scott HM. Insomnia treatment in New Zealand. N Z Med J*.* 2012;125(1349):46-59.

18. Scott GW, Scott HM, O'Keeffe KM, Gander PH. Insomnia - treatment pathways, costs and quality of life. Cost Eff Resour Alloc*.* 2011;9:10.

19. Lew JB, Simms K, Smith M, Lewis H, Neal H, Canfell K. Effectiveness modelling and economic evaluation of primary HPV screening for cervical cancer prevention in New Zealand. PLoS One*.* 2016;11(5):e0151619.

20. Friedman J, Reed P, Sharplin P, Kelly P. Primary prevention of pediatric abusive head trauma: a cost audit and cost-utility analysis. Child Abuse Negl*.* 2012;36(11-12):760-770.

21. Kelly P, Wilson K, Mowjood A, Friedman J, Reed P. Trialling a shaken baby syndrome prevention programme in the Auckland District Health Board. N Z Med J*.* 2016;129(1430):39-50.

22. Gander P, Scott G, Mihaere K, Scott H. Societal costs of obstructive sleep apnoea syndrome. N Z Med J*.* 2010;123(1321):13-23.

23. Lake RJ, Horn BJ, Dunn AH, Parris R, Green FT, McNickle DC. Cost-effectiveness of interventions to control Campylobacter in the New Zealand poultry meat food supply. J Food Prot*.* 2013;76(7):1161-1167.

24. Sears A, Baker MG, Wilson N, Marshall J, Muellner P, Campbell DM, Lake RJ, French NP. Marked campylobacteriosis decline after interventions aimed at poultry, New Zealand. Emerg Infect Dis*.* 2011;17(6):1007-1015.

25. Webb M, Fahimi S, Singh GM, Khatibzadeh S, Micha R, Powles J, Mozaffarian D. Cost effectiveness of a government supported policy strategy to decrease sodium intake: global analysis across 183 nations. BMJ*.* 2017;356:i6699.

26. Wilson N, Nghiem N, Eyles H, Mhurchu CN, Shields E, Cobiac LJ, Cleghorn CL, Blakely T. Modeling health gains and cost savings for ten dietary salt reduction targets. Nutr J*.* 2016;15:44.

27. Nghiem N, Blakely T, Cobiac LJ, Pearson AL, Wilson N. Health and economic impacts of eight different dietary salt reduction interventions. PLoS One*.* 2015;10(4):e0123915.

28. Maddison R, Pfaeffli L, Whittaker R, Stewart R, Kerr A, Jiang Y, Kira G, Leung W, Dalleck L, Carter K *et al*. A mobile phone intervention increases physical activity in people with cardiovascular disease: Results from the HEART randomized controlled trial. Eur J Prev Cardiolog*.* 2015;22(6):701-709.

29. Dalziel K, Segal L, Katz R. Cost-effectiveness of mandatory folate fortification v. other options for the prevention of neural tube defects: results from Australia and New Zealand. Public Health Nutr*.* 2010;13(4):566-578.

30. Tio M, Andrici J, Cox MR, Eslick GD. Folate intake and the risk of prostate cancer: a systematic review and meta-analysis. Prostate Cancer Prostatic Dis*.* 2014;17(3):213-219.

31. Sopina E, Ashton T. Cost-effectiveness of a cervical screening program with human papillomavirus vaccine. Int J Technol Assess Health Care*.* 2011;27(4):290-297.

32. Panattoni L, Brown PM, Te Ao B, Webster M, Gladding P. The cost effectiveness of genetic testing for CYP2C19 variants to guide thienopyridine treatment in patients with acute coronary syndromes: a New Zealand evaluation. Pharmacoeconomics*.* 2012;30(11):1067-1084.

33. Simms KT, Smith MA, Lew JB, Kitchener HC, Castle PE, Canfell K. Will cervical screening remain cost-effective in women offered the next generation nonavalent HPV vaccine? Results for four developed countries. Int J Cancer*.* 2016;139(12):2771-2780.

34. Te Ao B, Brown P, Fink J, Vivian M, Feigin V. Potential gains and costs from increasing access to thrombolysis for acute ischemic stroke patients in New Zealand hospitals. Int J Stroke*.* 2015;10(6):903-910.

35. Te Ao BJ, Brown PM, Feigin VL, Anderson CS. Are stroke units cost effective? Evidence from a New Zealand stroke incidence and population-based study. Int J Stroke*.* 2012;7(8):623-630.

36. Keall MD, Pierse N, Howden-Chapman P, Cunningham C, Cunningham M, Guria J, Baker MG. Home modifications to reduce injuries from falls in the Home Injury Prevention Intervention (HIPI) study: a cluster-randomised controlled trial. Lancet*.* 2015;385(9964):231-238.

37. Wilson N, Kvizhinadze G, Pega F, Nair N, Blakely T. Home modification to reduce falls at a health district level: Modeling health gain, health inequalities and health costs. PLoS One*.* 2017;12(9):e0184538.

38. Pega F, Kvizhinadze G, Blakely T, Atkinson J, Wilson N. Home safety assessment and modification to reduce injurious falls in community-dwelling older adults: cost-utility and equity analysis. Inj Prev*.* 2016;22:420-426.

39. Milne RJ, Hockey H, Rea H. Long-term air humidification therapy is cost-effective for patients with moderate or severe chronic obstructive pulmonary disease or bronchiectasis. Value Health*.* 2014;17(4):320-327.

40. Rush E, Obolonkin V, McLennan S, Graham D, Harris JD, Mernagh P, Weston AR. Lifetime cost effectiveness of a through-school nutrition and physical programme: Project Energize. Obes Res Clin Pract*.* 2014;8(2):e115-122.

41. Pinto D, Robertson MC, Abbott JH, Hansen P, Campbell AJ, Team MOAT. Manual therapy, exercise therapy, or both, in addition to usual care, for osteoarthritis of the hip or knee. 2: economic evaluation alongside a randomized controlled trial. Osteoarthritis Cartilage*.* 2013;21(10):1504-1513.

42. Carrasco LR, Lee VJ, Chen MI, Matchar DB, Thompson JP, Cook AR. Strategies for antiviral stockpiling for future influenza pandemics: a global epidemic-economic perspective. J R Soc Interface*.* 2011;8(62):1307-1313.

43. Saunders-Hastings P, Reisman J, Krewski D. Assessing the State of Knowledge Regarding the Effectiveness of Interventions to Contain Pandemic Influenza Transmission: A Systematic Review and Narrative Synthesis. PLoS One*.* 2016;11(12):e0168262.

44. Heneghan CJ, Onakpoya I, Jones MA, Doshi P, Del Mar CB, Hama R, Thompson MJ, Spencer EA, Mahtani KR, Nunan D *et al*. Neuraminidase inhibitors for influenza: a systematic review and meta-analysis of regulatory and mortality data. Health Technol Assess*.* 2016;20(42):1-242.

45. Jefferson T, Jones MA, Doshi P, Del Mar CB, Hama R, Thompson MJ, Onakpoya I, Heneghan CJ. Risk of bias in industry-funded oseltamivir trials: comparison of core reports versus full clinical study reports. BMJ Open*.* 2014;4(9):e005253.

46. Jefferson T, Jones MA, Doshi P, Del Mar CB, Hama R, Thompson MJ, Spencer EA, Onakpoya I, Mahtani KR, Nunan D *et al*. Neuraminidase inhibitors for preventing and treating influenza in healthy adults and children. Cochrane Database Syst Rev*.* 2014(4):CD008965.

47. Harris A, Cooper BA, Li JJ, Bulfone L, Branley P, Collins JF, Craig JC, Fraenkel MB, Johnson DW, Kesselhut J *et al*. Cost-effectiveness of initiating dialysis early: a randomized controlled trial. Am J Kidney Dis*.* 2011;57(5):707-715.

48. Leung W, Roberts V, Gordon LG, Bullen C, McRobbie H, Prapavessis H, Jiang Y, Maddison R. Economic evaluation of an exercise-counselling intervention to enhance smoking cessation outcomes: The Fit2Quit trial. Tob Induc Dis*.* 2017;15 (1) (no pagination)(21).

49. Arnold EP, Milne DJ, English S. Conservative treatment for incontinence in women in rest home care in Christchurch: Outcomes and cost. Neurourol Urodyn*.* 2016;35(5):636-641.

50. Boyd M, Baker MG, Mansoor OD, Kvizhinadze G, Wilson N. Protecting an island nation from extreme pandemic threats: Proof-of-concept around border closure as an intervention. PLoS One*.* 2017;12(6):e0178732.

51. Duncan GE. Determining the health benefits of poultry industry compliance measures: the case of campylobacteriosis regulation in New Zealand. N Z Med J*.* 2014;127(1391):22-37.

52. Foley L, Maddison R, Jones Z, Brown P, Davys A. Comparison of two modes of delivery of an exercise prescription scheme. N Z Med J*.* 2011;124(1338):44-54.

53. Fyfe C, Borman B, Scott G, Birks S. A cost effectiveness analysis of community water fluoridation in New Zealand. N Z Med J*.* 2015;128(1427):38-46.

54. Herd DW, Newbury C, Brown PM. Cost benefit analysis of amethocaine (Ametop) compared with EMLA for intravenous cannulation in a children's emergency department. Emerg Med J*.* 2010;27(6):456-460.

55. Keall MD, Newstead S. An evaluation of costs and benefits of a vehicle periodic inspection scheme with six-monthly inspections compared to annual inspections. Accid Anal Prev*.* 2013;58:81-87.

56. Lee S, Knox A, Zeng IS, Coomarasamy C, Blacklock H, Issa S. Primary prophylaxis with granulocyte colony-stimulating factor (GCSF) reduces the incidence of febrile neutropenia in patients with non-Hodgkin lymphoma (NHL) receiving CHOP chemotherapy treatment without adversely affecting their quality of life: cost-benefit and quality of life analysis. Support Care Cancer*.* 2013;21(3):841-846.

57. Lemanu DP, Singh PP, Berridge K, Burr M, Birch C, Babor R, MacCormick AD, Arroll B, Hill AG. Randomized clinical trial of enhanced recovery versus standard care after laparoscopic sleeve gastrectomy. Br J Surg*.* 2013;100(4):482-489.

58. Macmillan A, Connor J, Witten K, Kearns R, Rees D, Woodward A. The societal costs and benefits of commuter bicycling: simulating the effects of specific policies using system dynamics modeling. Environ Health Perspect*.* 2014;122(4):335-344.

59. McAuley KA, Taylor RW, Farmer VL, Hansen P, Williams SM, Booker CS, Mann JI. Economic evaluation of a community-based obesity prevention program in children: the APPLE project. Obesity*.* 2010;18(1):131-136.

60. Sammour T, Zargar-Shoshtari K, Bhat A, Kahokehr A, Hill AG. A programme of Enhanced Recovery After Surgery (ERAS) is a cost-effective intervention in elective colonic surgery. N Z Med J*.* 2010;123(1319):61-70.
